# Secondary infections in critically ill patients with COVID-19

**DOI:** 10.1186/s13054-021-03672-9

**Published:** 2021-08-31

**Authors:** Giacomo Grasselli, Emanuele Cattaneo, Gaetano Florio

**Affiliations:** 1 Department of Anesthesia, Intensive Care and Emergency, Fondazione IRCCS Ca’ Granda Ospedale Maggiore Policlinico, Milan, Italy; 2 Department of Pathophysiology and Transplantation, University of Milan, Milan, Italy

## Abstract

This article is one of ten reviews selected from the Annual Update in Intensive Care and Emergency Medicine 2021. Other selected articles can be found online at https://www.biomedcentral.com/collections/annualupdate2021. Further information about the Annual Update in Intensive Care and Emergency Medicine is available from https://link.springer.com/bookseries/8901.

## Introduction

Since December 2019, when the first case of human transmission of the severe acute respiratory syndrome coronavirus 2 (SARS-CoV-2) was reported in Wuhan (China), more than a hundred million confirmed cases of coronavirus disease 2019 (COVID-19) have been described worldwide, and the pandemic declared on March 11, 2020 by the World Health Organization is still ongoing.

The clinical spectrum of SARS-CoV-2 infection ranges from asymptomatic disease to severe disease requiring hospitalization and admission to the intensive care unit (ICU) [1]. Recent multicenter studies showed that 5–32% of hospitalized patients with COVID-19 needed ICU admission [2–5], mainly for acute respiratory distress syndrome (ARDS) requiring endotracheal intubation and invasive mechanical ventilation [2–4, 6, 7]. According to the available published data, the mortality of critically ill patients with COVID-19 ranges from 16 to 78% [3, 6–8].

For a number of reasons, patients with COVID-19 admitted to the ICU are at high risk of developing infectious complications during their ICU stay. First, they frequently develop multiple organ failure with need for vasopressors, renal replacement therapy (RRT) and, in some cases, extracorporeal membrane oxygenation support. The duration of mechanical ventilation and the ICU lengths of stay of these patients are therefore usually prolonged (up to 19 days for mechanical ventilation and up to 49 days for ICU length of stay [5, 9]). Second, COVID-19 *per se* is associated with significant dysfunction of the patient’s immune system. Multiple studies have shown the involvement of both innate and acquired immunity as a response to SARS-CoV-2 infection. Preliminary Chinese studies detected a reduction in both CD4+ T and CD8+ T lymphocyte counts, an increase in neutrophils and a reduction in interferon gamma (IFN-γ) serum concentrations [10, 11]. Further studies confirmed these findings and showed a cytokine pattern characterized by excess pro-inflammatory molecules (cytokine storm [12]), inhibition of natural killer cells (NK and NKT) and cytotoxic lymphocytes, and morphological and phenotypical alterations of monocytes [13–15]. Third, after the publication of the results of the RECOVERY trial [16], treatment with systemic corticosteroids has become standard of care in all patients requiring supplemental oxygen. In addition, a number of drugs aimed at blunting the immune system response to the viral infection (for example cytokine inhibitors [tocilizumab, anakinra, sarilumab] or complement inhibitors [eculizumab]) are frequently administered to these patients and several trials are ongoing to assess their efficacy. Finally, secondary bacterial and fungal infections as a complication of viral respiratory diseases have been described during previous pandemics (2002 severe acute respiratory syndrome [SARS] [17], 2009 swine influenza pandemic [18], and 2012 Middle East respiratory syndrome [MERS] [19]) and some studies have highlighted their role in increasing the severity of the viral pneumonia [18].

A recent review of the literature showed that the incidence of co-infections (i.e., infections detected at admission) in patients with COVID-19 is less than in previous pandemics [20]. Data on secondary infections (i.e., infections acquired during the course of ICU stay) are scarce. The aim of the present chapter is to summarize the available evidence on the epidemiology, risk factors, impact on outcome and principles of treatment of secondary infections in critically ill patients with COVID-19

## Epidemiology and risk factors

### Bacterial infections

In patients with H1N1 influenza, the incidence of bacterial infections complicating the course of the viral pneumonia is 25–50% and they are associated with increased duration of mechanical ventilation, prolonged ICU stay and increased mortality [21]. For these reasons, early diagnosis and adequate management are mandatory in critically ill patients. As mentioned above, limited data are available on secondary bacterial infections in patients hospitalized for SARS-CoV-2 infection. A recent review reports a low incidence of bacterial or fungal infections in hospitalized COVID-19 patients, ranging from 6 to 15%, but most of the cited studies were conducted in China and included patients admitted mainly to ordinary wards and not to the ICU; hence, these data cannot be extrapolated to the population of critically ill patients admitted to the ICU in western countries.

The most common bacterial complication of COVID-19 is ventilator-associated lower respiratory tract infection (VA-LRTI), which includes ventilator-associated pneumonia (VAP) and ventilator-associated tracheobronchitis. The mechanism underlying bacterial co-infection in viral pneumonia is damage to the ciliated cells, which leads to impaired mucociliary clearance and increased adhesion of bacteria to mucins, resulting in enhanced bacterial colonization of the airways [22]. In addition to these mechanisms, other risk factors for bacterial secondary infections typical of ICU patients are the presence of ARDS and the prolonged duration of mechanical ventilation [23]. A recent multicenter European study described the cumulative incidence of VA-LRTI in patients with COVID-19 admitted to the ICU compared to patients with other viral and non-viral pneumonias. The overall incidence of VA-LRTI was 50%, significantly higher than in the other two groups, despite the fact that patients with SARS-CoV-2 pneumonia had lower severity scores (Simplified Acute Physiology Score [SAPS II] and Sequential Organ Failure Assessment [SOFA] score) at ICU admission and fewer comorbidities [24]. This finding has been confirmed by two other studies. The first evaluated the incidence of VAP in critically ill COVID-19 patients in the UK and showed that COVID-19 patients were significantly more likely to develop VAP than patients without COVID-19 [25]. The second study was a multicenter, observational trial conducted in several European countries and described the clinical characteristics of 4244 critically ill COVID-19 patients; the incidence of VAP in intubated patients was 58% [9]. As previously mentioned, prolonged duration of mechanical ventilation and a high incidence of ARDS, both typical of COVID-19, together with the administration of drugs affecting immune system function (in the multicenter European study 37.3% of the patients were treated with steroids) certainly contribute to this increased risk of secondary respiratory infections.

The most common bacteria involved in VA-LRTI in COVID-19 patients are Gram-negative bacilli, mainly *Pseudomonas aeruginosa*, *Enterobacter* spp. and *Escherichia coli*, followed by Gram-positive cocci, mainly *Staphylococcus aureus* [24]. Notably, some smaller reports describe different microorganism prevalence. For example, Sharifipour et al. [26] reported that among 19 ICU patients, secondary respiratory infections were caused by *Acinetobacter baumannii* in 90% of the cases and *S. aureus* in the remaining 10%. However, the findings of these small, single-center case series are clearly influenced by the local epidemiology and are not representative of the general population of COVID-19 ICU patients.

The second most common secondary infections in critically ill COVID-19 patients are bloodstream infections (BSI). An Italian report estimated a cumulative risk of developing an episode of BSI of nearly 25% after 15 days of ICU stay and higher than 50% after 30 days. In multivariable analysis, anti-inflammatory treatment with tocilizumab or with methylprednisolone was independently associated with the development of BSI [27]. Buetti et al. [28] conducted a case control study comparing BSIs in 235 COVID-19 and 235 non-COVID 19 patients admitted to the ICU in France and described incidences of 14.9% and 3.4%, respectively. In patients infected with SARS-CoV-2, BSIs occurred a median of 12 days after ICU admission. The most common microorganisms responsible for BSIs were coagulase-negative staphylococci (36%). The authors also observed a significant increase in the risk of BSIs in COVID-19 patients treated with tocilizumab or anakinra [28] (Table 1).

**Table 1:** Characteristics and main findings of the studies describing secondary infections in patients with COVID-19

Study [ref]	Sample size	Setting	Incidence of secondary infections, %	Type and site of infection (%)	Microorganisms isolated (%)
Giacobbe et al. [27]	78	ICU	40	BSI (100)	Coag-neg staphylococci (24) *E. faecalis* (18) *S. aureus* (13)
He et al. [46]	918	Hospital	7	Pneumonia (32) BSI (25) UTI (22)	Coag-staphylococci (28) *A. baumannii* (21) *P. aeruginosa* (14)
Sharifipour et al. [26]	19	ICU	100^a^	VAP (100)	*A. baumannii* (90) *S. aureus* (10)
Fu et al. [47]	36	ICU	14	VAP (100)	*S. mantophilia* (40)
Li et al. [48]	1495	Hospital	7	Pneumonia (86) BSI (34) UTI (8)	*A. baumannii* (36) *K. pneumoniae* (31) *S. mantophilia* (6)
Rouzé et al. [24]	568	ICU	51	VAP (71) VAT (29)	*P. aeruginosa* (22) *Enterobacter* spp. (18) *S. aureus* (12)
Buetti et al. [28]	321	ICU	15	BSI	Coag-staphylococci (36) *Enterobacterales* (13) *P. aeruginosa* (13)
Dudoignon et al. [49]	54	ICU	37	VAP (75)	*P. aeruginosa* (33) *Enterobacteriaceae* (33) *S. aureus* (20)
Ripa et al. [50]	731	Hospital	9	BSI (85) LRTI (32)	Coag-staphylococci 70% of BSI *A. baumannii* 30% of LRTI

*BSI* bloodstream infection, *UTI* urinary tract infection, *VAP* ventilator associated pneumonia, *VAT* ventilator associated tracheobronchitis, *LRTI* lower respiratory tract infection

^a^Only patients who developed secondary infections were included in this study

### Fungal infections

It is well known that viral pneumonia caused by influenza virus can facilitate the development of invasive pulmonary aspergillosis, especially in patients presenting with ARDS, with a marked impact on the duration of hospitalization and mortality [29]. The limited data available in critically ill patients with COVID-19 seem to confirm the association between SARS-CoV-2 infection and development of invasive aspergillosis and some authors have suggested the existence of a clinical entity called COVID-19-associated pulmonary aspergillosis [30]. Risk factors for invasive pulmonary aspergillosis in COVID-19 patients are the direct lung damage due to the viral infection, use of corticosteroids, ARDS at presentation, treatment with broad-spectrum antibiotics, and comorbidities [30]. The initial reports from China were very heterogeneous, describing an incidence of infection with *Aspergillus* ranging from 3% up to 23% among critically ill patients with COVID-19 [31, 32]. This variability could be due to the lack of precise definition criteria and of a standardized diagnostic algorithm for invasive pulmonary aspergillosis, possibly resulting in the underestimation of the real incidence of invasive pulmonary aspergillosis in some studies, while in others the misinterpretation of colonization may have led to an overestimation of the risk. European studies report a high rate (from 20% to 35%) of invasive pulmonary aspergillosis among critical patients with ARDS due to COVID-19, with a high mortality rate, ranging from 45 to 67% [33, 34]. The most common *Aspergillus* spp. responsible for invasive pulmonary aspergillosis in these patients seems to be *Aspergillus fumigatus* (isolated in 90% of the cultures), followed by *Aspergillus flavus* [30].

## Diagnosis of secondary infections

### Bacterial infections

As previously mentioned, the most common secondary infections in critically ill patients with COVID-19 are VAP and BSIs. The diagnosis is made when the patient shows clinical symptoms and signs of infection and a new pathogen is detected in a biological specimen.

VAP is defined as the association of persistent pulmonary infiltrates on radiological imaging and positive microbiological cultures from a lower respiratory tract specimen with clinical suspicion of new onset pneumonia in a patient that has received at least 48 h of invasive mechanical ventilation [35–37]. Scores, such as the Clinical Pulmonary Infection Score (CPIS) [38] (based on six variables: temperature, blood leukocytes, aspect of tracheal secretions, oxygenation, radiographic infiltrates, and Gram stain on tracheal aspirates), have been developed to help clinicians diagnose VAP, but the most recent guidelines [36, 39] highlight the role of clinical signs of infection (i.e., new onset of fever, purulent secretion from the air-way, leukocytosis or leukopenia, worsening of blood oxygenation, increased need for inotropic and vasoactive agents) rather than the use of a score. Imaging techniques, such as chest X-ray [37], chest computed tomography, and, more recently, lung ultrasound [40, 41], tailored to detect new pulmonary infiltrates, and markers of inflammation (e.g., C-reactive protein, procalcitonin) can support the clinical diagnosis.

Adequate and specific antibiotic therapy, however, requires a microbiological diagnosis based on culture examinations and tests (e.g., Gram stain, biomarkers, rapid diagnostic assay, polymerase chain reaction [PCR]) to enable identification of the involved bacteria. Samples can be obtained from the distal airway in a more invasive way using bronchoscopy (i.e., bronchoalveolar lavage [BAL], protected specimen brush [PSB]), in a ‘less-invasive’ way (i.e., blind mini-BAL, blind PSB) or from the proximal airway (endotracheal aspirate); a recent meta-analysis [42] comparing cultures from proximal and distal airways showed no differences in patient outcome, but it should be remembered that sampling from the distal airway may be associated with an increased risk for the patient (i.e., hypoxemia, bleeding). Furthermore, invasive procedures are associated with potential exposure to aerosolized viral particles, which represents a risk for healthcare personnel.

BSI in critically ill patients is defined as the onset of signs and symptoms of infection within 24 h of a positive blood culture. Blood cultures and identification of specific bacteria represent the gold standard for the diagnosis, but a single positive culture is not suggestive of infection when a typical human skin contaminant is involved; in this case, the diagnosis requires at least two positive blood cultures for the microorganism within 48 h.

### Fungal infections

COVID-19-associated pulmonary aspergillosis should be suspected in all patients with COVID-19 who present with refractory fever lasting more than 3 days after an initial 48-h period of defervescence (following appropriate antibiotic therapy), worsening of gas exchange, onset of hemoptysis, or new pleural rubs [29]. A complete and accurate algorithm for diagnosing COVID-19-associated pulmonary aspergillosis is still lacking but it would be useful to search for *Aspergillus* spp. in respiratory samples (e.g., bronchoalveolar lavage, tracheal aspirate) and to use serologic biomarkers such as galactomannan on respiratory samples and serum. Other tests that may help in diagnosing COVID-19-associated pulmonary aspergillosis are aspergillus PCR and serum (1→3)-β-d-glucan.

## Principles of treatment

### Bacterial infections

The initial Surviving Sepsis Campaign guidelines for the management of critically ill patients with COVID-19 suggested an empiric antibacterial agent in all mechanically ventilated patients [43]. However, subsequent data have shown that, at ICU admission, patients infected with SARS-CoV-2 seldom have concomitant bacterial infection. For this reason, and because of the high incidence of infectious complications caused by multidrug-resistant (MDR) germs, most experts agree that prophylactic administration of an empiric antibiotic therapy in the absence of clear signs of a co-infection or of a secondary infection should be discouraged. Indeed, it has been demonstrated that inappropriate initial antimicrobial treatment is associated with increased mortality in VAP and with increased bacterial resistance [44]. In addition, in critically ill patients, different doses from those usually recommended may be used, either because normal doses may not achieve effective drug concentrations at the target site or because they can be associated with adverse reactions due to toxic concentrations. For these reasons, therapeutic drug monitoring of plasma trough levels is recommended.

Available guidelines [35, 36] recommend that empirical therapy should be started as soon as VAP is clinically suspected. The empirical therapy should be modified based on the results of the culture tests. The choice of the empirical treatment is based mainly on the patient’s risk factors for MDR pathogens, and on the local pattern of antimicrobial susceptibility. Among the risk factors for MDR is ARDS prior to VAP and hospital stay > 5 days, both very likely to be present in COVID-19 patients. In this case, the empirical treatment of choice should be a broad-spectrum anti-pseudomonas β-lactam plus a non-β-lactam antipseudomonal agent (e.g., piperacillin-tazobactam plus amikacin). When choosing the antibiotic, it is important to consider the local pattern of susceptibility, and the results of microbiological surveillance for patient colonization. Empiric coverage of methicillin-resistant *S. aureus* (MRSA) should be considered in units where the incidence of VAP is higher than 20% [35]. Once culture and susceptibility results are obtained, the main goal should be to remove unnecessary antibiotics (especially anti-MRSA and carbapenems) and use a narrow spectrum agent if possible.

### Fungal infections

Patients with invasive aspergillosis often have many comorbidities that, together with the underlying disease, can affect the pharmacokinetics of antifungal medications. As reported earlier for antibacterial agents, even for antimycotics the risk of not reaching the target concentration at the infection site or of toxicity exists, especially in critically ill patients, thus therapeutic drug monitoring is recommended. Given the high mortality rate of patients with critical COVID-19 and concomitant invasive pulmonary aspergillosis, treatment should be started as soon as the diagnosis of invasive pulmonary aspergillosis is made.

Voriconazole is recommended as first line treatment in invasive pulmonary aspergillosis, with a target plasma trough concentration of 2–6 mg/l. Repeated monitoring is indicated until steady-state level is confirmed or if there is a change in the patient’s clinical condition or suspected toxicity. In patients with liver dysfunction or when voriconazole cannot be administered, liposomal amphotericin B is appropriate. In patients who do not respond or do not tolerate initial therapy an echino-candin alone or in combination with voriconazole is indicated [45].

## Conclusion

Secondary infections, frequently caused by MDR germs, are common in critically ill patients with COVID-19 admitted to the ICU, as a result of a number of favoring conditions. Early and accurate diagnosis and institution of adequate antimicrobial treatment are essential to improve patient outcome. Preliminary published data indicate that secondary infections are associated with increased duration of mechanical ventilation and of ICU stay, and that they may have an impact on patient survival. However, data from large, well-designed studies are needed to confirm these findings and to improve our knowledge of the epidemiology and treatment of infections complicating the clinical course of COVID-19.

## Data Availability

All data generated or analyzed during this study are included in this published article (and its supplementary information files).
